# YK-4-250, a synthetic telmisartan–tempol conjugate: crystal structure of a stabilized free radical containing an angiotensin AT_1_ receptor inhibitor

**DOI:** 10.1107/S2053229626007242

**Published:** 2026-08-18

**Authors:** Simon Friedrich, Yali Kong, Faik N. Musayev, Milton L. Brown, B. Frank Gupton

**Affiliations:** a Department of Chemical and Life Science Engineering, Virginia Commonwealth University, Richmond, VA 23220, USA; b Department of Biomedical and Translational Sciences Macon & Joan Brock Virginia Health Sciences at Old Dominion University Norfolk VA 23507 USA; c Department of Medicinal Chemistry, School of Pharmacy, Virginia Commonwealth University, Richmond, VA 23298, USA; d Department of Medicine Macon & Joan Brock Virginia Health Sciences at Old Dominion University Norfolk VA 23501 USA; e Medicines for All Institute, Virginia Commonwealth University, Richmond, VA 23284, USA; University of Notre Dame, USA

**Keywords:** angiotensin receptor blocker, ARB, radiation mitigator, crystal structure, solid-state characterization, radical stabilization

## Abstract

We report the first crystal structure of the telmisartan–tempol conjugate 2,2,6,6-tetra­methyl-1-(λ′-oxidane­yl)piperidin-4-yl 4′-[(1,7′-dimethyl-2′-propyl-1*H*,3′*H*-[2,5′-bibenzo[*d*]imidazol]-3′-yl)meth­yl]-[1,1′-biphen­yl]-2-carboxyl­ate free radical (YK-4-250, C_42_H_46_N_5_O_3_), a dual-function inhibitor designed to target both the angiotensin II type 1 receptor (AT_1_R) and reactive oxygen species (ROS). Structural analysis reveals a unique crystal architecture in which the nitroxide radical is stabilized within a sterically protected environment while preserving the telmisartan pharmacophore essential for high-affinity receptor antagonism. YK-4-250 is a novel small-mol­ecule conjugate of telmisartan and tempol that incorporates a catalytic stabilized nitroxide radical and was rationally designed to mitigate gastrointestinal acute radiation syndrome (GI-ARS).

## Abstract 2

The first crystal structure of the telmisartan–tempol conjugate YK-4-250 is reported, revealing the intra­molecular C–H⋯π inter­actions that stabilize the nitroxide radical while preserving the angiotensin AT_1_ receptor antagonist pharmacophore.

## Introduction

The development of a small-mol­ecule mitigator for radiation-induced tissue injury remains a significant unmet need in both clinical and national defense applications. High-dose exposure to ionizing radiation from radiological accidents or nuclear detonations triggers com­plex multi-organ pathologies. While the hematopoietic system is highly vulnerable, exposure to higher doses ranging from 6 to 10 Gy induces gastrointestinal acute radiation syndrome (GI-ARS) (Freeman, 2025 ▸). The lethal pathology of GI-ARS manifests rapidly, driven by the profound ablation of highly proliferative intestinal epithelial stem cells (IESCs) within the crypts of Lieberkühn, coupled with severe microvascular endothelial damage (Paris *et al.*, 2001 ▸; Shaker & Rubin, 2010 ▸). This mucosal barrier breakdown leads to structural villus blunting, severe diarrhea, systemic sepsis, and death, typically benchmarked within 10 to 30 days in a partial-body irradiation (PBI) LD50/30 model (Kumar *et al.*, 2026 ▸). While medical countermeasures exist to support bone marrow recovery (Bunin *et al.*, 2023 ▸), there remains a critical therapeutic void for orally available small-mol­ecule countermeasures capable of mitigating GI-ARS when administered 24 h or longer post-exposure.

A mounting body of evidence highlights a lethal synergistic feed-forward loop between the local renin–angiotensin system (RAS) and chronic oxidative stress in driving this radiation-induced tissue injury (Kumar *et al.*, 2024 ▸; DiCarlo *et al.*, 2025 ▸). Ionizing radiation initiates immediate radiolytic water cleavage, generating a massive burst of primary reactive oxygen species (ROS). However, the long-term propagation of tissue damage is sustained by chronic metabolic ROS production. This sustained oxidative stress is largely mediated by the upregulation of Angiotensin II (Ang II) and its subsequent binding to the Angiotensin II Type 1 receptor (AT_1_R) (Fan *et al.*, 2023 ▸).

Activation of the AT_1_R by Ang II triggers the membrane-bound enzyme com­plex NADPH oxidase (Nox), dramatically amplifying intra­cellular superoxide (O_2_^**.**–^) generation (Garrido *et al.*, 2009 ▸). This secondary wave of ROS induces persistent mitochondrial dysfunction, lipid peroxidation of endothelial membranes, and pro-inflammatory signalling *via* nuclear factor kappa beta (NFkb). In the gastrointestinal tract, this cascade accelerates endothelial cell apoptosis, deprives the intestinal crypts of necessary perfusion, and halts crypt regeneration (Orzechowska-Licari *et al.*, 2022 ▸). Conversely, elevated ROS levels further upregulate local AT_1_R expression (Bhatt *et al.*, 2014 ▸) and stimulate local Ang II production (Dikalov & Naza­rewicz, 2013 ▸), locking the irradiated GI microenvironment into a chronic cycle of inflammation, vaso­constriction, and fibroproliferative remodeling (Nguyen Dinh Cat *et al.*, 2013 ▸). To inter­cept this destructive signalling loop, YK-4-250 was rationally designed as a synthetic conjugate of telmisartan, a clinically approved AT_1_R antagonist, and tempol, a stable nitroxide radical with well-established anti­oxidant and radioprotective properties (Kumar *et al.*, 2026 ▸). The com­pound blocks radiation-induced Ang II sig­nalling at the AT_1_R while simultaneously scavenging ROS generated by radiolysis of water and activation of NADPH oxidase (Nox).

The physiological rationale for this specific hybrid design exploits critical mechanistic synergies to achieve what monotherapy cannot. By blocking the AT_1_R, the telmisartan core prevents the initial Ang II-dependent assembly and activation of Nox, cutting off the primary enzymatic source of secondary metabolic ROS. Concurrently, the integrated nitroxide tempol moiety acts as a highly efficient intra­cellular superoxide dismutase (SOD) mimetic and per­oxy­nitrite scavenger, rapidly detoxifying residual O_2_^**.**–^ and preventing the formation of highly destructive hydroxyl radicals (Wilcox, 2010 ▸). This dual mechanism simultaneously relieves AT_1_R-mediated microvascular vasoconstriction and protects the thin-walled endothelial cells of the villi from oxidative apoptosis, thereby preserving the sub-epithelial vascular niche essential for regenerating intestinal stem cells (Kumar *et al.*, 2026 ▸). Furthermore, telmisartan offers a distinct pharmacological of extended half-life of nearly 22 hours, which works in tandem to extend the half-life of tempol’s anti­oxidant property to downregulate pro-inflammatory cytokines (TNF-a, IL-1b) and dampen the systemic inflammatory response accom­panying barrier breakdown.

Crucially, this bifunctional strategy addresses the severe pharmacokinetic limitations that have historically hindered free anti­oxidants from clinical translation. While native anti­oxidant proteins or free nitroxides suffer from poor oral bioavailability and ultra-short biological half-lives, the lipophilic telmisartan core serves as an effective pharmacological vehicle. This scaffold optimizes gastrointestinal absorption and tissue distribution, yielding the first orally available small-mol­ecule mitigator engineered to break the AT_1_R–ROS feed-forward loop directly within the injured mesenteric and intestinal mucosa after exposure to partial body irradiation (PBI) LD50/30. The integration of these two pharmacophores, AT_1_R antagonism and catalytic anti­oxidation, represents a dual-mechanism strategy for mitigating GI-ARS. Here, we report the first single-crystal X-ray structure of a telmisartan–tempol conjugate, providing structural insight into its mol­ecular geometry, radical-stabilizing inter­actions, and conformational features that may underlie its biological activity and support its development as a potential active pharmaceutical ingredient (API) for GI-ARS.

## Experimental

### Synthesis and crystallization

**Figure 1:**
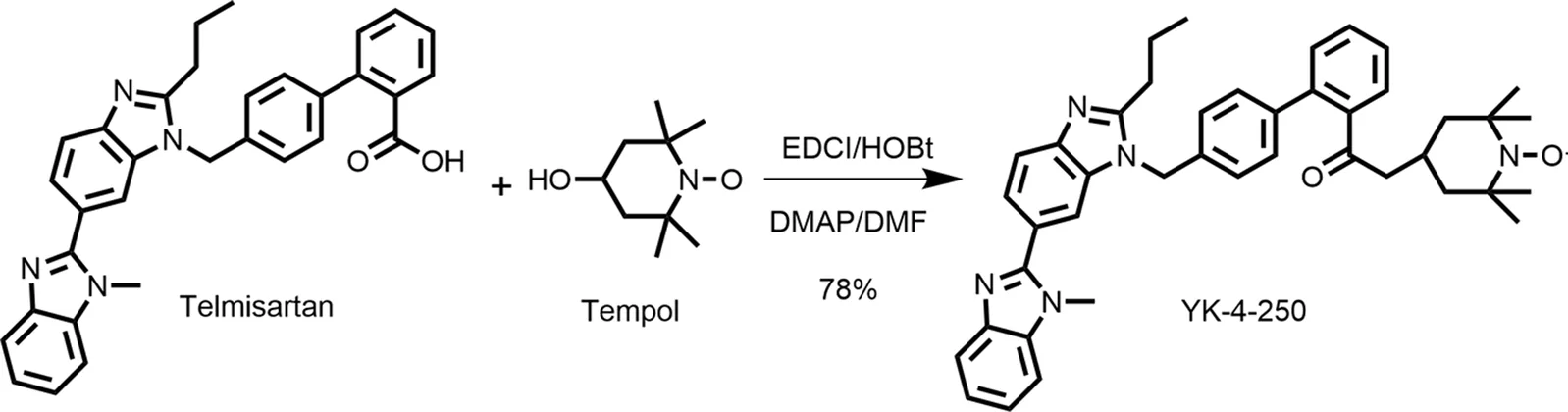
The reaction scheme for the preparation of YK-4-250.

YK-4-250 was synthesized by the coupling of telmisartan and tempol under EDCI/HOBt coupling conditions in DMF at room tem­per­a­ture for 12 h. The product was purified by nor­mal-phase flash chromatography using methyl­ene chlo­ride–methanol as eluent to give analytically pure material in 78% yield (Fig. 1 ▸). Structure characterization was per­for­med by HRMS (TOF): calculated for C_42_H_47_N_5_O_3_ (*M* + H)^+^: 669.3679; found: 669.3675. Single crystals suitable for X-ray diffraction were obtained by slow evaporation of a 1:1 (*v*/*v*) ethyl acetate/hexa­nes solution over 7 d at room tem­per­a­ture.

### Refinement

Crystal data, data collection and structure refinement details are summarized in Table 1 ▸. H atoms bonded to C atoms were placed in calculated positions and refined using a riding model, with *U*_iso_(H) = 1.2*U*_eq_(C) for aromatic/methyl­ene groups and 1.2*U*_eq_(C) for methyl groups. The C—H bond distances were set to *de facto* standard values corresponding to their respective hybridization states.

### Simultaneous differential scanning calorimetry (DSC)/thermogravimetric analysis (TGA)

**Figure 2:**
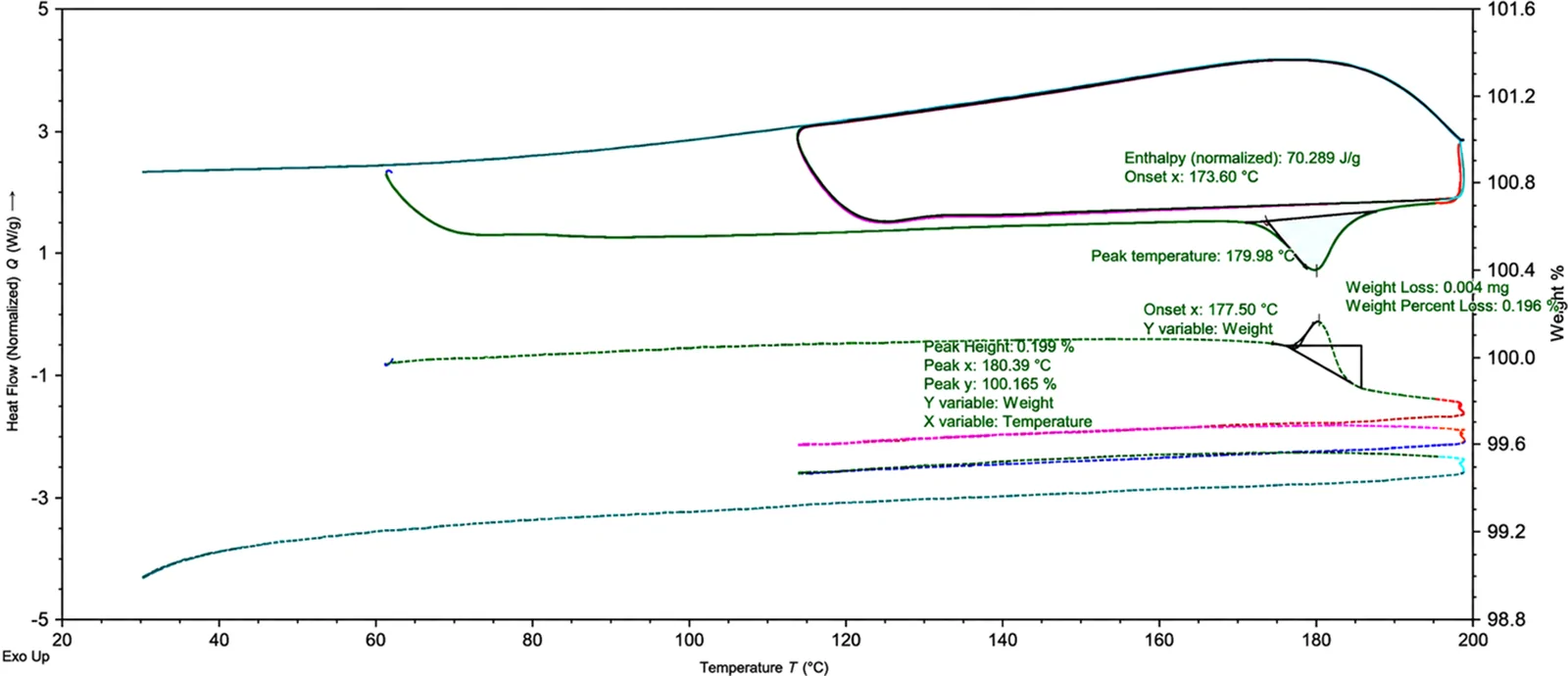
(Top) DSC trace of three cycles 388.15–471.15 K and (bottom) TGA curve of three cycles 388.15–471.15 K.

Differential scanning calorimetry (DSC) is widely regarded as a key technique for inferring the thermodynamic relationships between multiple crystal forms (Yu, 1995 ▸). Simultaneous DSC/TGA (SDT) was performed on an SDT650 to supplement the obtained DSC data with any mass change events. A single ∼2.5 mg crystal of YK-4-250 was added to an aluminium DSC pan without a sealing lid. The chamber was held inert by steady N_2_ gas purge while the chamber was ramped to 471.15 K at a rate of 5 K min^−1^. Three cycles were programmed to cool to 388.15 K and subsequently heated to 471.15 K; this was done to capture any melting and fusion events and verify these events’ parameters. However, none were observed, a single exothermic event (70.3 J g^−1^) was observed at an onset of 446.75 K with a peak at 453.15 K. This event was accom­panied by a 0.2% weight loss (Fig. 2 ▸). It is hypothesized that the melting of the com­pound frees the radical and triggers an irreversible decom­position. Due to this, it was not possible to determine the thermodynamic stability of this structure or if other more thermodynamically stable conformations exist by DSC. Telmisartan is known to exhibit two polymorphs; therefore, it is pertinent to elucidate if this com­pound also exhibits polymorphism in future work (Cunha *et al.*, 2021 ▸; Dinnebier *et al.*, 2000 ▸).

### *ORCA* density functional theory (DFT) optimization of a single mol­ecule

The theoretical single-mol­ecule structure was pre-optimized with XTB (Dinnebier *et al.*, 2000 ▸) set to normal and fully optimized with *ORCA* (Version 6.0.1) using the B3LYP functional and def2-SVP basis set using TightSCF convergence; the radical is specified *via* one unpaired electron designation (Neese, 2022 ▸; Bannwarth *et al.*, 2019 ▸).

## Results

### Structural commentary and DFT theoretical structure

YK-4-250 crystallizes in the monoclinic space group *Ia* with one mol­ecule in the asymmetric unit. The mol­ecule adopts an extended conformation in which the telmisartan and tempol fragments are oriented nearly perpendicular to each other.

**Figure 3:**
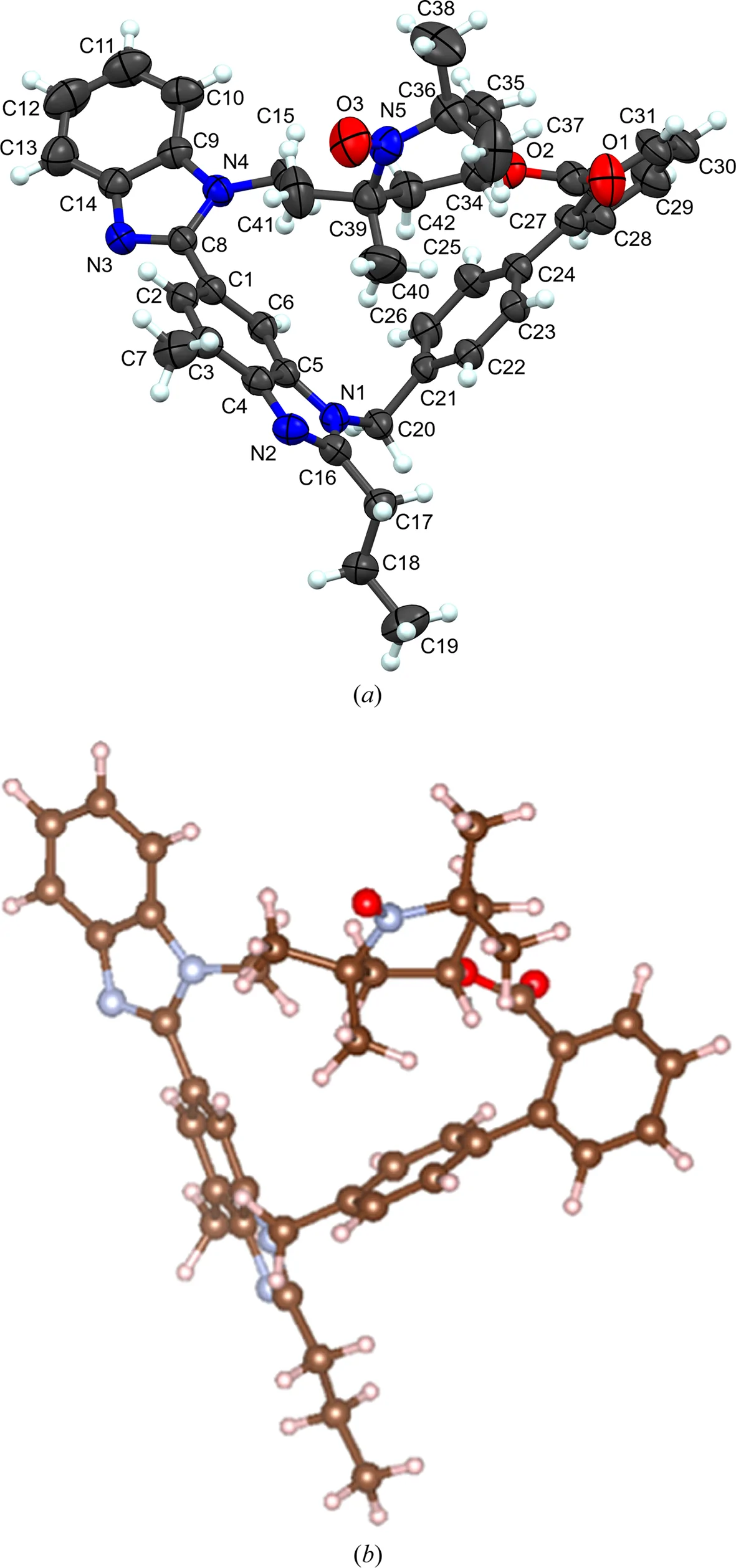
(*a*) Experimentally measured YK-4-250, drawn with 50% probability displacement ellipsoids, and (*b*) theoretically calculated YK-4-250 *via ORCA*.

Key bond lengths and angles in the telmisartan-derived biphenyl fragments match those in previously reported telmisartan structures; however, the dibenzimidazole inter­facial angle of YK-4-250 [Fig. 3 ▸(*a*)] is significantly more eclipsed than in telmisartan form A (Amano *et al.*, 2012 ▸; Cunha *et al.*, 2021 ▸; Dinnebier *et al.*, 2000 ▸; Singh *et al.*, 2024 ▸). The presence of the radical does not significantly distort the tempol backbone. The inter­facial angles of the experimentally calculated conformer’s biphenyl rings and dibenzimidazole system are listed and com­pared to the theoretically optimized single mol­ecule, as well as the known telmisartan form A in (see Table S1 in the supporting information) (Dinnebier *et al.*, 2000 ▸). The theoretical single-molecule structure [Fig. 3 ▸(*b*)] revealed that the radical is 94% confined to the nitroxide moiety, as calculated *via* total spin occupancy in the N—O system.

The com­parison of the experimentally obtained crystal structure to the optimized single-mol­ecule structure provides insight into the influence of crystal packing on mol­ecular conformation. The radical confinement to the nitroxide moiety is not meaningfully affected by the crystal structure, in both the gas-optimized single mol­ecule and crystal structure, 94% of the radical electron spin is confined to the N—O moiety. The dihedral (inter­facial) angles of the ring systems in the mol­ecule give insight into the inter­molecular inter­actions and intra­molecular conformation. The dihedral angle changes of the dibenzimidazole and biphenyl rings reflects the balance between inter­molecular inter­actions and intra­molecular conjugation. Biphenyl by itself in solution at room tem­per­a­ture exhibits a rather flexible dihedral angle in the range 32–45° (Eaton & Steele, 1973 ▸). The DFT-optimized single molecule exhibits a comparable interfacial angle of 47.3°, whereas the packed crystal shows a significant shift to 61.3°. Furthermore, the close intermolecular contact distance falls well within the sum of the respective atomic van der Waals radii, confirming that the observed packing is stabilized by standard van der Waals forces. For the dibenzimidazole rings, no exact analogue was found in the literature. However, an article by Antonov and co-workers described the dihedral angles of a 2′-phenyl-2,5′-bibenzodimidazole [2,5′-BBIm (c1)] to be 18.28° in the aqueous phase (Antonov *et al.*, 2022 ▸). For comparison, the DFT-optimized single molecule exhibits an interfacial angle of 37.9°, whereas the experimentally obtained crystal structure displays a reduced angle of 31.9°. This contraction in the solid state is likely driven by fewer intermolecular interactions involving the benzimidazole subunits.

### Supra­molecular features

In the extended lattice, mol­ecules pack in layers parallel to the *ab* plane (see Fig. S2 in the supporting information). Inter­molecular C—H⋯O contacts (3.1 Å) link mol­ecules into one-dimensional chains along the *b* axis. Additional stabilization is provided by three C—H⋯π inter­actions (Table 3 ▸) involving the phenyl and benzimidazole rings. The crystal structure exhibits mainly van der Waals inter­actions. The nitroxide radical does not participate in significant inter­molecular inter­actions, consistent with preserved radical stability in the solid state. In fact, the only near neighbors (<4 Å) are methyl or phenyl H atoms (Table 2 ▸) for the nearest 12 H-atom neighbors of the radical O atom.

### Database survey

A search of the Cambridge Structural Database (CSD, Version 5.46; Groom *et al.*, 2016 ▸) revealed no previously reported crystal structures of telmisartan–tempol conjugates or related stabilized nitroxide–AT_1_R inhibitor analogues. While crystal forms of telmisartan (Dinnebier *et al.*, 2000 ▸) and structural analogues of tempol (Cunha *et al.*, 2021 ▸) have been described independently, to our knowledge, YK-4-250 represents the first structurally characterized mol­ecule in which an AT_1_R antagonist is covalently linked to a stable nitroxide radical.

## Discussion

### Radical stabilization through crystal packing

The most significant finding is the structure by which the nitroxide radical in YK-4-250 is stabilized in the solid state. Unlike tempol derivatives (Cunha *et al.*, 2021 ▸), which typically rely on hy­dro­gen-bonding networks involving the nitroxide O atom, YK-4-250 presents no conventional hy­dro­gen-bond donors; both benzimidazole N atoms are substituted and no O—H or N—H groups are present, structurally precluding the participation of atom O3 in such inter­actions. Strong hy­dro­gen-bond donors would impose a thermodynamic driving force sufficient to position donors near O3 despite steric cost; their absence means the steric environment provided by the flanking dimethyl substituents becomes the sole determinant of access to the radical center. The result is a sterically protected environment in which only the adjacent CH_3_ groups and aromatic H atoms from neighboring YK-4-250 mol­ecules approach within 4 Å of O3, exclusively as weak C—H⋯O contacts (Table 2 ▸). This intra­molecular steric shielding, which restricts inter­molecular access to the radical center rather than relying on conventional hy­dro­gen bonding, may offer a promising design principle for developing stable radical-containing pharmaceuticals that must preserve their paramagnetic character during storage and formulation. B3LYP/def2-SVP spin population analysis in *ORCA* (Version 6.0.1) confirms 94% spin confinement to the N—O moiety, with negligible change between the gas-phase optimized and crystal-conformation geometries, indicating that radical localization is robust to the conformational constraints imposed by crystal packing.

### Crystal packing and solid-state organization

Unlike telmisartan form A, which achieves crystal cohesion through extensive hy­dro­gen bonding (Cunha *et al.*, 2021 ▸), YK-4-250 organizes its solid-state structure through a hierarchy of weaker inter­actions. At the primary level, inter­molecular C—H⋯O contacts (H⋯O = 3.1 Å) link mol­ecules into one-dimensional chains along the *b* axis, with these chains assembling into layers parallel to the *ab* plane. Three C—H⋯π inter­actions involving the phenyl and benzimidazole ring systems (Table 2 ▸) provide lateral cohesion between chains. Together these contacts define a packing arrangement in which directional weak inter­actions substitute collectively for the conventional hy­dro­gen-bonding networks present in related structures.

### Thermal behaviour and polymorph considerations

Thermal analysis revealed a single exothermic event at 446.75 K accom­panied by minimal mass loss (0.2%). This behavior renders conventional DSC-based polymorph screening unfeasible, leaving the question of whether additional thermodynamically stable forms exist. Given that telmisartan exhibits two known polymorphs (Dinnebier *et al.*, 2000 ▸) and the flexible ester linker at the tempol conjugation site introduces additional degrees of freedom, polymorph screening through solution-mediated approaches remains necessary to inform solid-form control during pharmaceutical development. Identifying the most stable polymorph and understanding transformation pathways will be critical for ensuring consistent manufacturing, long-term stability of this potential drug candidate, and FDA evaluation.

### Implications for structure–activity optimization and formulation development

The crystal structure provides a key insight for designing next-generation derivatives that balance receptor-binding potency with radical stabilization. Key design considerations include: (i) maintaining the conformation that preserves the active AT_1_R pharmacophore geometry, (ii) engineering intra­molecular inter­actions that stabilize the radical through spatial isolation, and (iii) minimizing inter­molecular contacts with the nitroxide center that could com­promise radical stability during processing or storage. The characterization of the solid-state properties of YK-4-250, including its packing behavior and thermal stability profile, informs formulation strategies for this promising radiation mitigator and supports the continued development as a dual-mechanism pharmaceutical agent for gastrointestinal acute radiation syndrome.

## Supplementary Material

Crystal structure: contains datablock(s) I, global. DOI: 10.1107/S2053229626007242/ov3185sup1.cif

Structure factors: contains datablock(s) I. DOI: 10.1107/S2053229626007242/ov3185Isup2.hkl

Additional figures and tables. DOI: 10.1107/S2053229626007242/ov3185sup3.pdf

CCDC reference: 2522559

## Figures and Tables

**Table 1:** Experimental details

Crystal data	
Chemical formula	C_42_H_46_N_5_O_3_
*M*_r_	668.84
Crystal system, space group	Monoclinic, *Ia*
Temperature (K)	295
*a*, *b*, *c* (Å)	9.4556 (1), 27.1981 (3), 15.0100 (2)
β (°)	106.828 (2)
*V* (Å^3^)	3694.89 (8)
*Z*	4
Radiation type	Cu *K*α
μ (mm^−1^)	0.60
Crystal size (mm)	0.65 × 0.38 × 0.20

Data collection	
Diffractometer	Rigaku XtaLAB AFC11
Absorption correction	Multi-scan (*CrysAlis PRO*; Rigaku OD, 2021 ▸)
*T*_min_, *T*_max_	0.550, 1.000
No. of measured, independent and observed [*I* > 2σ(*I*)] reflections	16498, 4987, 4906
*R*_int_	0.019
(sin θ/λ)_max_ (Å^−1^)	0.627

Refinement	
*R*[*F*^2^ > 2σ(*F*^2^)], *wR*(*F*^2^), *S*	0.029, 0.082, 1.06
No. of reflections	4987
No. of parameters	459
No. of restraints	2
H-atom treatment	H-atom parameters constrained
Δρ_max_, Δρ_min_ (e Å^−3^)	0.12, −0.11
Absolute structure	Flack *x* determined using 1302 quotients [(*I*^+^) − (*I*^−^)]/[(*I*^+^) + (*I*^−^)] (Parsons *et al.*, 2013 ▸)
Absolute structure parameter	0.06 (11)

Computer programs: *CrysAlis PRO* (Rigaku OD, 2021 ▸), *SHELXT* (Sheldrick, 2015*a* ▸), *SHELXL2016* (Sheldrick, 2015*b* ▸), and *OLEX2* (Dolomanov *et al.*, 2009 ▸).

**Table 2:** List of the nearest 12 H-atom neighbors of the radical O atom The H atoms belonging to the dimethyl sub-units adjacent to the N—O moiety are marked in bold. Angles are included for O3⋯H—C.

Atom pair	Distance (Å)	Angle (°)
**O3⋯H41*A***	**2.45**	**96.50**
**O3⋯H38*C***	**2.53**	**92.80**
O3⋯H15*B*	2.53	158.03
**O3⋯H40*C***	**2.63**	**99.00**
**O3⋯H37*A***	**2.65**	**98.20**
O3⋯H29	2.76	155.42
**O3⋯H38*A***	**2.88**	**72.40**
O3⋯H10	2.92	161.74
**O3⋯H41*C***	**2.97**	**68.30**
**O3⋯H37*C***	**3.29**	**60.40**
**O3⋯H40*A***	**3.30**	**60.00**
O3⋯H15*C*	3.65	70.06

**Table 3:** List of the C—H⋯*Cg* (*Cg* is a ring centroid) distances and the corresponding C—H⋯*Cg* angles *Cg*1 is the centroid of the N3/C8/N4/C9/C14 ring, *Cg*2 that of the C21–C26 ring, and *Cg*3 that of the C1–C6 ring.

C—H⋯*Cg*	Distance (Å)	Angle (°)
C13—H13⋯*Cg*1	2.90	164.40
C2—H2⋯*Cg*2	2.98	158.84
C41—H41B⋯*Cg*3	2.99	171.64
