# Supplementary material for: YK-4-250, a synthetic telmisartan–tempol conjugate: crystal structure of a stabilized free radical containing an angiotensin AT1 receptor inhibitor
Source: Acta Crystallogr C Struct Chem. 2026 Aug 18;82(Pt 9):547–52. doi: 10.1107/S2053229626007242 (PMC13543282; doi:10.1107/S2053229626007242)
Supplement: Supplementary file 1 [file c-82-00547-sup3.pdf]

STRUCTURAL CHEMISTRY

Volume 82 (2026)

Supporting information for article:

YK-4-250, a synthetic telmisartan–tempol conjugate: crystal structure of a stabilized free radical containing angiotensin AT_1 receptor inhibitor

Simon Friedrich, Yali Kong, Faik N. Musayev, Milton L. Brown and B. Frank Gupto

Crystallographic data (CIF) and structure-factor file have been deposited at the Cambridge Crystallographic Data Centre (Deposition no. CCDC-2522559). This material is available free of charge via http://www.ccdc.cam.ac.uk/structures/.

[redacted]

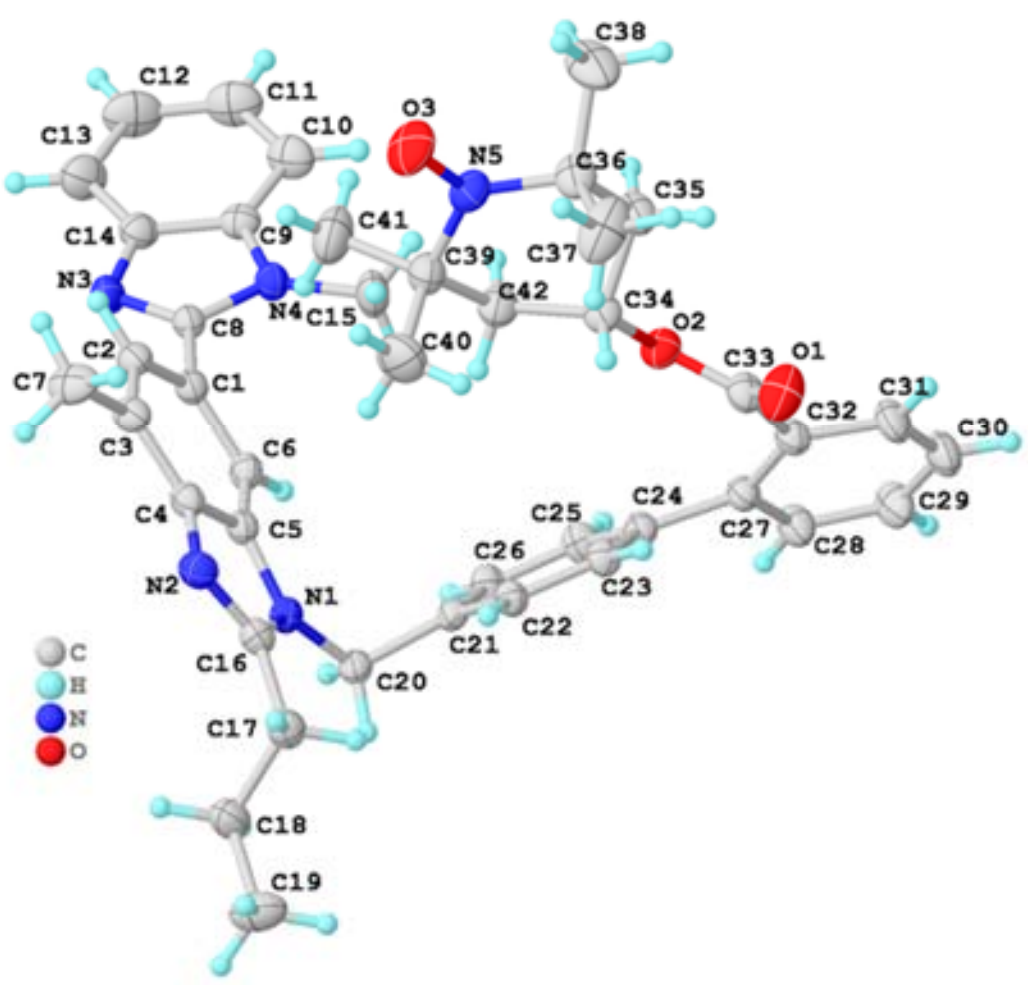

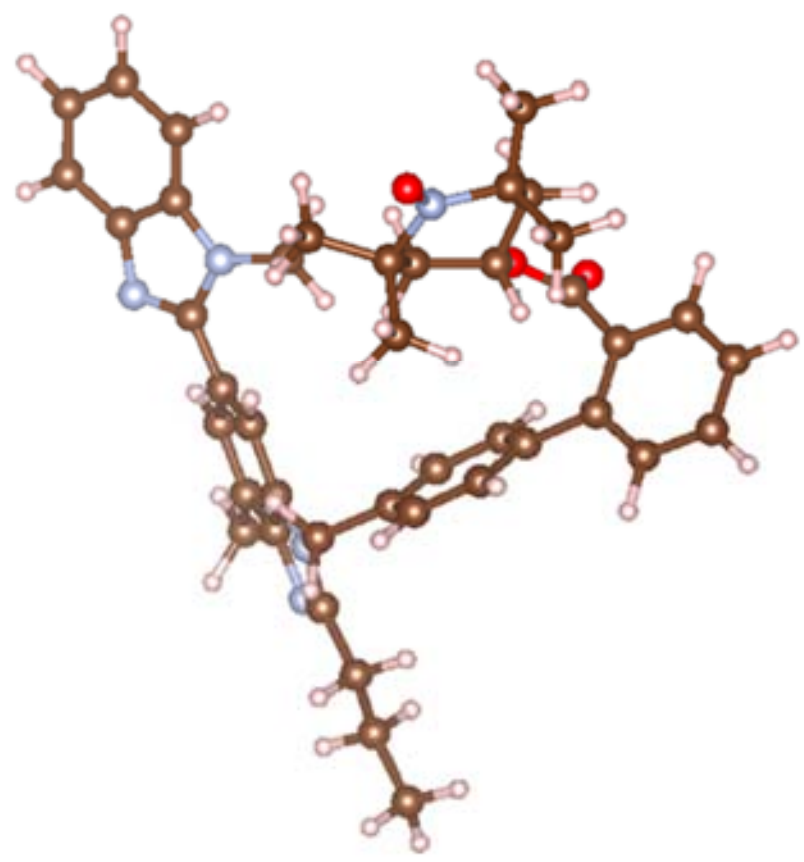

S1. Synthesis scheme of YK-4-250 (top); Experimental with 50 % displacement ellipsoids(left); DFT optimized single molecule (right).

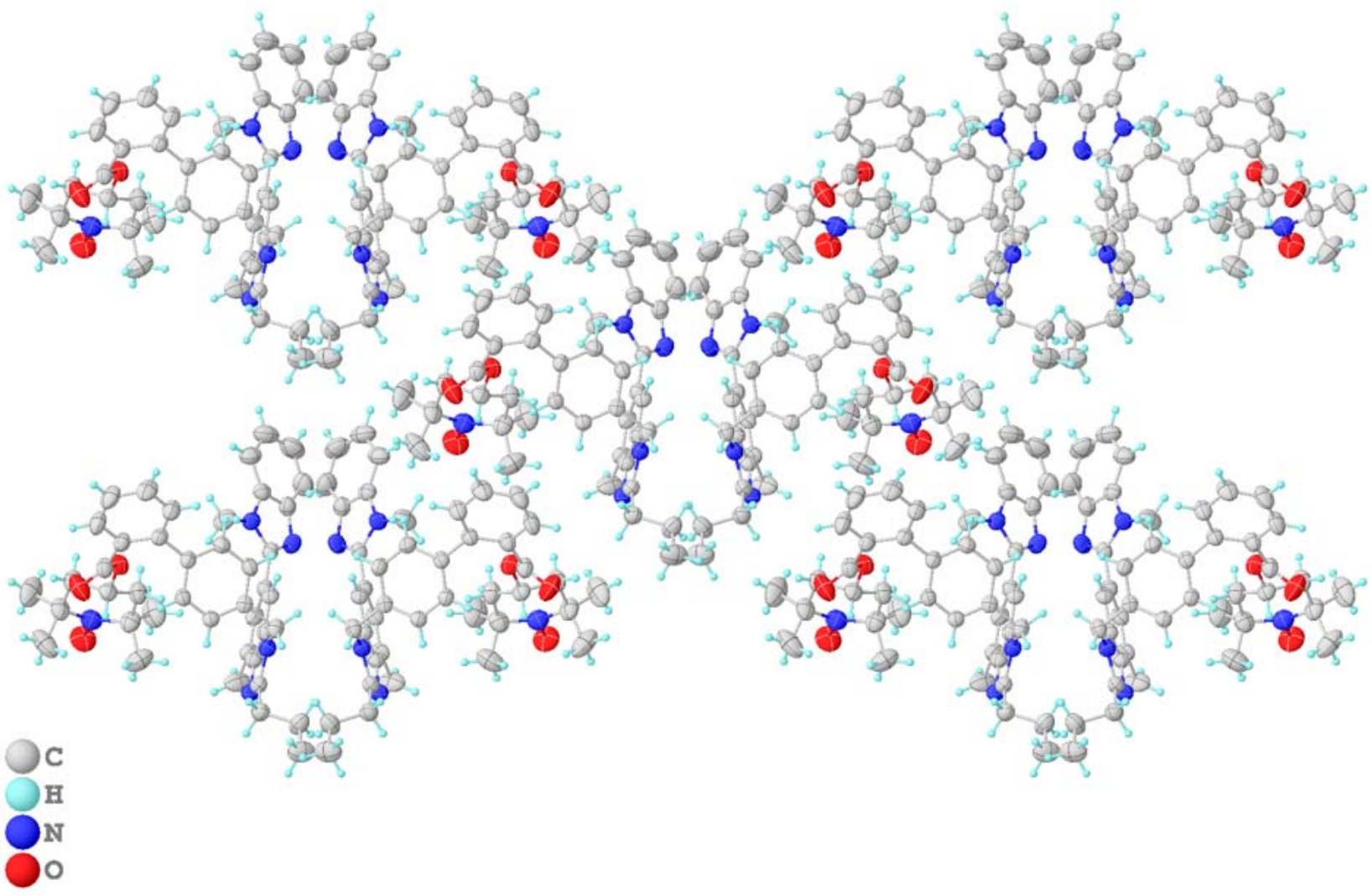

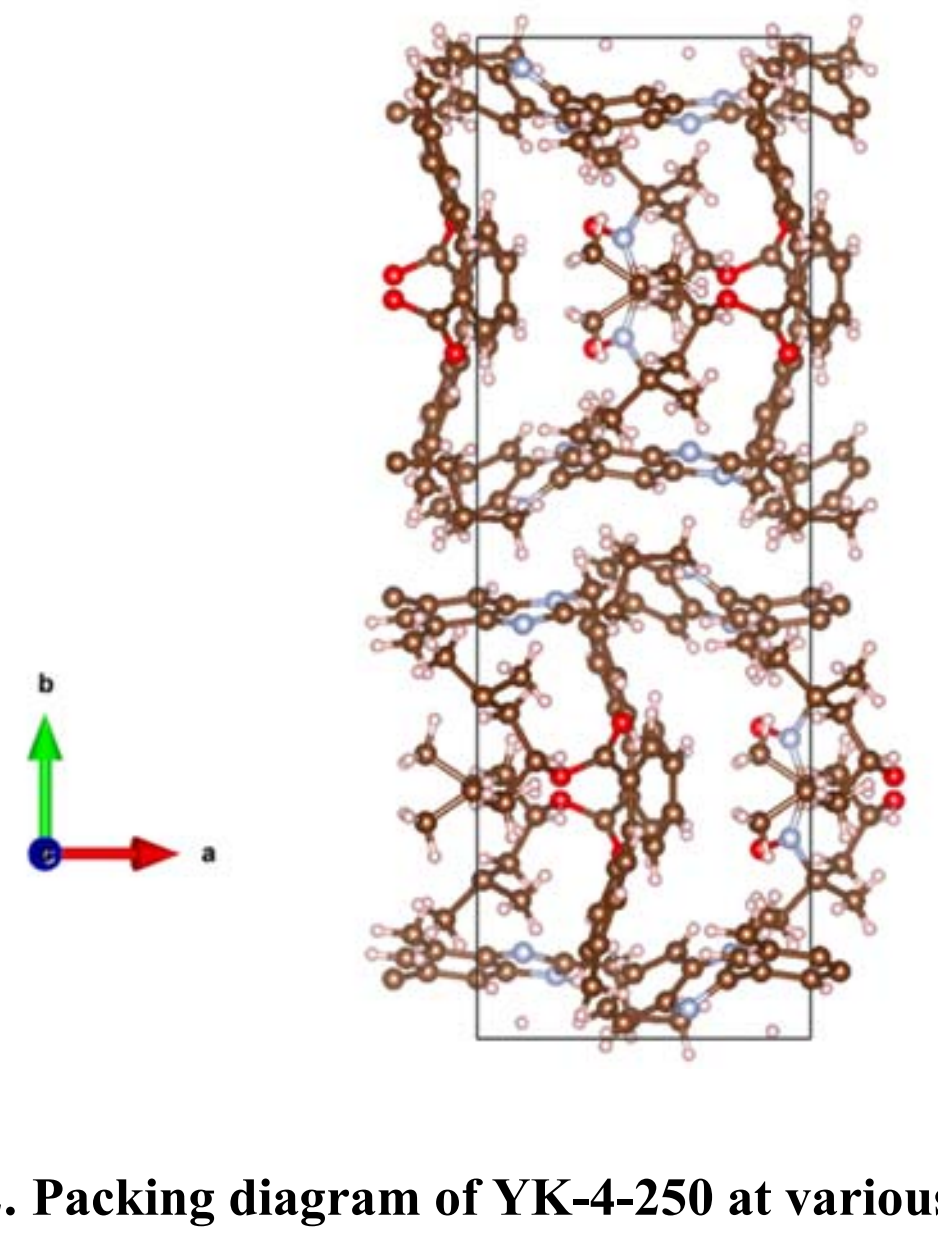

S2. Packing diagram of YK-4-250 at various angles

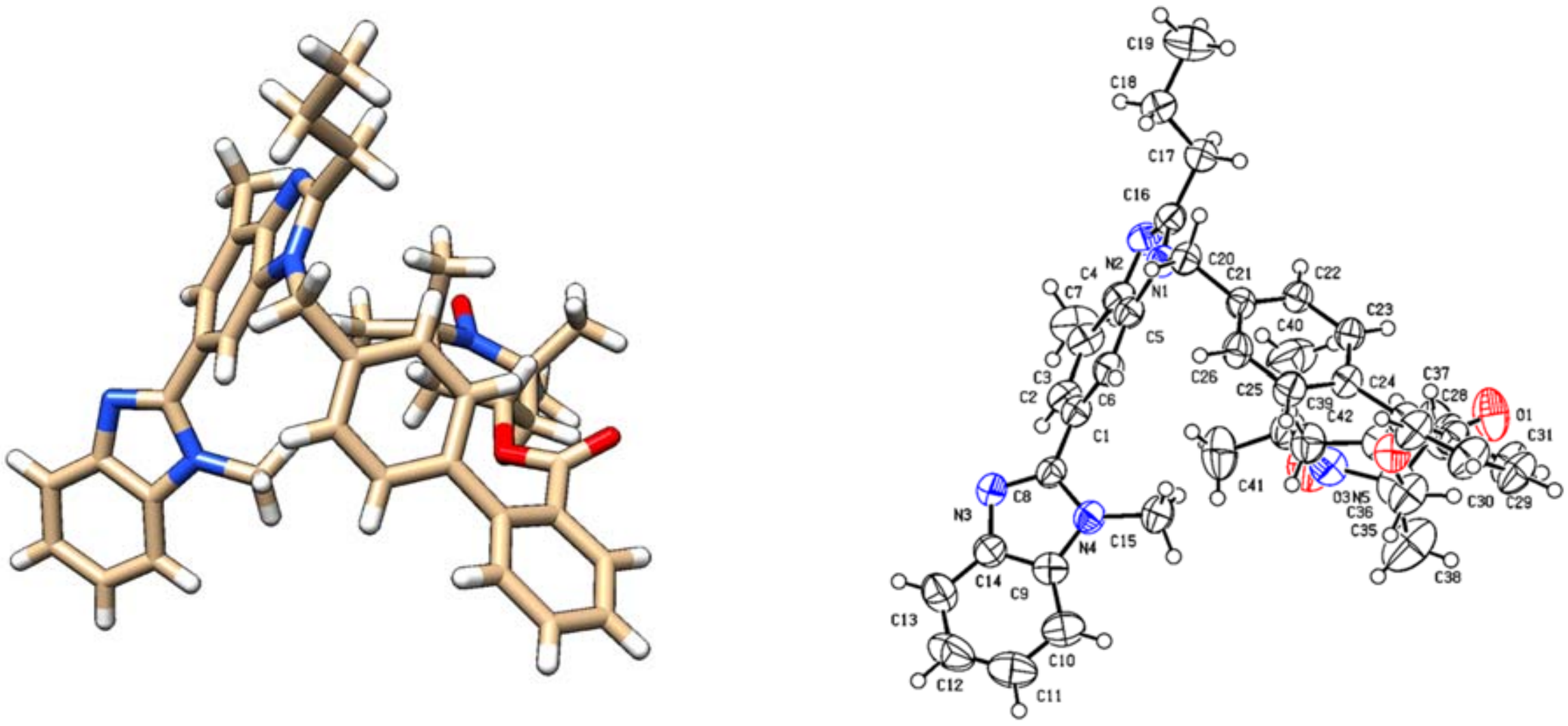

S3. X-ray crystal structure of YK-4-250 and its molecular atom arrangement

Table S1 Interfacial angles of the rings within experimentally determined crystallite of YK-4-250 compared to the theoretical single molecule & Telmisartan Form A.

Structure/Sub-unit	Biphenyl	Di-Benzimidazole
Experimental – Crystal	61.3	31.9
Theoretical – Single Molecule	47.3	37.9
Literature – Telmisartan (Form A) [5]	66.8*	50.9*

*Angles were calculated from the CCDC deposited .cif file listed in (Dinnebier *et al*., 2000).

Table S2 List of the nearest 12 hydrogen neighbours of the O-radical atom, in bold are hydrogens belonging to the dimethyl sub-units adjacent to the N-O moiety.

Atom Pair	Distance (Å)
**(O46-H49A)**	**2.45**
**(H50C-O46)**	**2.53**
(O46-H19B)	2.53
**(O46-H48C)**	**2.63**

**(O46-H47A)**	**2.65**
(O46-H31)	2.76
**(O46-H50A)**	**2.88**
(O46-H13)	2.92
**(H49C-O46)**	**2.97**
**(O46-H47C)**	**3.29**
**(O46-H48A)**	**3.30**
(O46-H19C)	3.65
